# Genomic characterization of tigecycline-resistant *Escherichia coli* and *Klebsiella pneumoniae* isolates from hospital sewage

**DOI:** 10.3389/fmicb.2023.1282988

**Published:** 2023-11-10

**Authors:** Ying Li, Yu Fu, Yichuan Qiu, Qian Liu, Ming Yin, Luhua Zhang

**Affiliations:** ^1^The School of Basic Medical Science and Public Center of Experimental Technology, Southwest Medical University, Luzhou, Sichuan, China; ^2^Department of Clinical Laboratory, Hospital of Chengdu Office of People’s Government of Tibetan Autonomous Region, Chengdu, Sichuan, China; ^3^Department of Clinical Laboratory, The Affiliated Traditional Chinese Medicine Hospital of Southwest Medical University, Luzhou, Sichuan, China

**Keywords:** *tet*(X4), *tmexCD-toprJ*, tigecycline resistance, plasmid, efflux pumps

## Abstract

**Introduction:**

The tigecycline-resistant Enterobacterales have emerged as a great public concern, and the mobile *tet*(X) variants and *tmexCD-toprJ* efflux pump are mainly responsible for the spread of tigecycline resistance. Hospital sewage is considered as an important reservoir of antimicrobial resistance, while tigecycline resistance in this niche is under-researched.

**Methods:**

In this study, five *Escherichia coli* and six *Klebsiella pneumoniae* strains were selected from a collection of tigecycline-resistant Enterobacterales for further investigation by antimicrobial susceptibility testing, conjugation, whole-genome sequencing, and bioinformatics analysis.

**Results:**

All five *E. coli* strains harbored *tet*(X4), which was located on different plasmids, including a novel IncC/IncFIA(HI1)/IncHI1A/IncHI1B(R27) hybrid structure. In addition, *tet*(X4)-bearing plasmids were able to transfer by conjugation and be stabilized in the recipient in the absence of antibiotics. *tmexCD1-toprJ1* was identified in two *K. pneumoniae* (LZSFT39 and LZSRT3) and it was carried by a novel multidrug-resistance transposon, designated Tn*7368*, on a novel IncR/IncU hybrid plasmid. In addition, we found that two *K. pneumoniae* (LZSFZT3 and LZSRT3) showed overexpression of efflux genes *acrB* and *oqxB*, respectively, which was most likely to be caused by mutations in *ramR* and *oqxR*.

**Discussion:**

In conclusion, the findings in this study expand our knowledge of the genetic elements that carry tigecycline resistance genes, which establishes a baseline for investigating the structure diversity and evolutionary trajectories of human, animal, and environmental tigecycline resistomes.

## 1. Introduction

Antibiotic resistance poses a serious threat to global public health. Tigecycline, the third-generation tetracycline antibiotic, is a last-resort drug to treat serious infections, especially those caused by carbapenem-resistant Enterobacterales and *Acinetobacter* spp., which have been classified as critical-priority bacteria by the World Health Organization (Tacconelli et al., 2018). Tigecycline resistance has emerged over the years, while the mechanism is complex and has not yet been fully elucidated. Previous studies have reported that overexpression of efflux pumps (such as AcrAB and OqxAB; Sheng et al., 2014; He et al., 2015), or mutation in the *tet*(A) gene (coding for Tet(A) efflux protein) (Xu et al., 2021) often underlies the resistance mechanisms of tigecycline. High-level expression of *acrAB* can result from the up-regulation of *ramA*, which can be caused by a mutation in *ramR*, a local transcriptional repressor of *ramA* (Wang et al., 2015)*. rarA* overexpression upregulates the *oqxAB* efflux pump while *oqxR*, a transcriptional repressor, can downregulate the *oqxAB* (Veleba et al., 2012). In addition, the mutation in the *rpsJ* (encoding the S10 ribosomal subunit; Beabout et al., 2015), might be also involved in tigecycline resistance among *Klebsiella pneumoniae* isolates.

*tet*(X) has been shown to encode a flavin-dependent monooxygenase that degrades tigecycline (Forsberg et al., 2015). The recent emergence and dissemination of plasmid-mediated *tet*(X) variants [*tet*(X3)-*tet*(X6)] that confers high-level tigecycline resistance raise the concern that the efficacy of this last-resort antibiotic may be compromised, further limiting clinical treatment choices. Among them, *tet*(X3) and *tet*(X4) are frequently detected in Acinetobacter and Enterobacterales isolates from different origins, especially animals, and their meat products in China (Sun et al., 2019, 2020; Chen et al., 2020). In addition to *tet*(X) genes, the emergence of plasmid-borne RND-type efflux pump *tmexCD-toprJ* gene clusters that confers resistance to multiple drugs, including tigecycline, also poses a huge risk to public health. Six *tmexCD-toprJ* variants have been identified in different bacterial species, and they are frequently detected in food-producing animals (Wang C. Z. et al., 2021; Wang Q. et al., 2021; Gao et al., 2022; Liu et al., 2022; Wang C. et al., 2023; Wang J. et al., 2023). Data from nationwide surveillance in China showed that *tmexCD-toprJ*-positive bacteria are rare (0.64%, 48/7517) in clinical settings, with *Pseudomonas* and *Klebsiella* serving as the main reservoir of *tmexCD-toprJ* variants (Dong et al., 2022).

Plasmid-mediated horizontal gene transfer plays a vital role in the dissemination of tigecycline resistance. *Tet*(X4) has been reported to be carried on plasmids with a variety of replicon types, including IncQ1, IncX1, IncHI1, IncFIB, and untypeable plasmids (Fang et al., 2019; Sun et al., 2020; Yu et al., 2021), and the mobilization of *tet*(X4) seems to be closely related to IS*CR2* (He et al., 2019). IncR, IncFIA, IncFIB, and IncFIB/IncHI1B hybrid plasmids are mainly vectors for efflux pump gene cluster *tmexCD-toprJ*, and different mobile elements, including site-specific integrases and IS elements, such as IS*26* and IS*6100*, might facilitate its transmission (Peng et al., 2021; Dong et al., 2022). Although food animals are the principal sources of mobile tigecycline resistance determinates, it is also crucial to monitor the expanding antibiotic resistance in the environment under the ‘One Health’ framework, particularly in hospital sewage, as it is a hotspot for horizontal gene transfer for antimicrobial resistance genes (ARGs) between bacterial communities, and also a reflection of the ARGs and pathogens that are prevalent in the hospital (Cahill et al., 2019). A recent investigation demonstrated that *tmexCD-toprJ* coexists with *mcr-3* and carbapenemase genes in bacteria in hospital sewage in Zhejiang province in China, which highlighted the potential risks of antimicrobial resistance development and spread in water systems (Wu et al., 2023). Yet reports are currently lacking on the prevalence and genetic characteristics of tigecycline resistance in hospital sewage.

In the present study, we aimed to determine the genetic contexts of tigecycline resistance genes and diversity of resistance plasmids in six tigecycline-resistant *Escherichia coli* and eight tigecycline-resistant *K. pneumoniae* isolates from hospital sewage in Southwest China, where the prevalence of tigecycline resistance has rarely been reported (Wu et al., 2023).

## 2. Materials and methods

### 2.1. Bacterial isolates

Six samples were collected from the influx of the wastewater treatment plant of three tertiary care hospitals, the Affiliated Hospital of Southwest Medical University (2,200 beds), the Affiliated Traditional Chinese Medicine Hospital of Southwest Medical University (3,000 beds), Luzhou People’s Hospital (1,000 beds)in Luzhou City, Sichuan province, Southwest China in 2021. Each hospital was sampled twice at 3-month intervals. These three hospitals are located in the north, east and middle of the city, respectively, and account for most of the city’s medical care. Sewage samples (5 mL) were collected at a depth of ~10 cm below the water surface using a sterile centrifuge tube during weekdays in the morning (9 am to 11 am). The samples were stored on ice before being taken to the laboratory for subsequent testing within the following 1 h. After sufficient mixing, 100 μL of water sample were spread on MacConkey agar supplemented with 2 μg/mL tigecycline before overnight incubation at 37°C. One to three colonies of each type with different colors and morphology from each sample were picked. Species identification of the colonies was performed by partial amplification and sequencing of the 16 s rRNA gene as described previously with the primes 27F/1492R (Table 1; Lane, 1991). All the isolates were examined for the presence of *tet*(X) variants, and *tmexCD-toprJ* variants by PCR using specific primers (Table 1). All the PCR products were analyzed on 1.5% agarose gel by electrophoresis. The positive PCR products were determined using Sanger sequencing by Tsingke Bioinformatics Technology Co. Ltd. (Beijing, China) and were compared with reported sequences by BLASTn.^1^

**Table 1:** Primers used in this study.

Primer name	Sequence (5′-3′)	Annealing temperature	Product size	Reference
27F	AGAGTTTGATCCTGGCTCAG	53°C	~1,450 bp	Lane (1991)
1492R	ACGGCTACCTTGTTACGACTT			
*tet*(X3)-F	GACACTTGATCTGCACAGGGATT	53°C	685 bp	Ji et al. (2020)
*tet*(X3)-R	CCCTACAAAAGATGATGTCAAAC			
*tet*(X4)-F	CTGATTCGTGTGACATCATCTTTTG	53°C	204 bp	Ji et al. (2020)
*tet*(X4)-F	GTTAAATTTCCCATTGGTCAGATTA			
*tet*(X5)-F	GGTATCAACATTTCAATGCTTG	53°C	265 bp	Ji et al. (2020)
*tet*(X5)-F	CGATTCGTCCTGCGTATCTTTTG			
*tet*(X6)-F	AAACCGAGTGAAACAGCAGA	53°C	363 bp	This study
*tet*(X6)-R	TTCTTTGTAGCGTTCATCCC			
*tmexD*-F	CAGCCAGGACTACAACTTC	53°C	1,314 bp	Gao et al. (2022)
*tmexD*-R	TAGAGGAACTTCGGATTGC			
*acrB*-F	GAAAGTGCTGGATGAGATGACGAAT	60°C	174 bp	Li et al. (2023a)
*acrB*-R	GCTTCAACTTTGTTTTCCTCACCCG			
*acrE*-F	ATGCCTCCGTGATG	60°C	175 bp	Li et al. (2023a)
*acrE*-R	TCCGCTTCCGCTTTGA			
*oqxB*-F	ATCAGGCGCAGGTTCAGGT	60°C	200 bp	Li et al. (2023a)
*oqxB*-R	CGCCAGCTCATCCTTCACTT			
*ramA*-F	CGAGTGGATTGATGATAACC	60°C	194 bp	Li et al. (2023a)
*ramA*-R	TATCGTAGACCCGCTGAT			
*rarA*-F	GTTTGTTGACGAAGTGCA	60°C	327 bp	He et al. (2015)
*rarA*-R	GCCATCATTTCCAGGGTA			
16 s-F	TGATCATGGCTCAGATTGAACG	60°C	120 bp	This study
16 s-R	GCAGTTTCCCAGACATTACTCAC			

### 2.2. Susceptibility to antibiotics

Antimicrobial susceptibility testing was performed using the Kirby Bauer disk diffusion method on Mueller-Hinton agar as recommended by Clinical and Laboratory Standards Institute (CLSI) and interpreted according to CLSI guidelines (CLSI, 2023). The following antibiotics were tested: amikacin (AMK, 30 μg), gentamicin (GEN, 10 μg), tetracycline (TET, 30 μg), ciprofloxacin (CIP, 5 μg), tigecycline (TIG, 15 μg), cefoxitin (FOX, 30 μg), chloramphenicol (CHL, 30 μg), cefotaxime (CTX, 30 μg), meropenem (MEM, 10 μg), sulfamethoxazole-trimethoprim (SXT, 25 μg). The diameter of inhibition zones was interpreted following the CLSI recommendations for Enterobacteriaceae. *E. coli* ATCC 25922 served as the control strain. MICs of tigecycline was determined using the microdilution broth method and interpreted according to the FDA criteria (susceptible, ≤2 μg/mL; intermediate, 4 μg/mL; resistant, ≥8 μg/mL).^2^

### 2.3. Genomic DNA sequencing and data analysis

Genomic DNA of selected strains was extracted using Rapid Bacterial Genomic DNA Isolation Kit (Sangon Biotech, Shanghai, China) according to the manufacturer’s protocol. Purified DNA was subjected to short-read sequencing on an Illumina HiSeq 2000 platform (Illumina, San Diego, CA, United States) with the 150-bp paired-end approach by the Tsingke Biotech (Beijing, China). Clean reads were *de novo* assembled into contigs using SPAdes with the careful mode (Bankevich et al., 2012). Four isolates (LZSFT34, LZSFT39, LZSFZT33, and LZSRT11) were additionally sequenced on the long-read MinION sequencer (Nanopore, Oxford, United Kingdom). Both the long MinION reads and short Illumina reads were *de novo* assembled by using Unicycler v0.4.3 (Wick et al., 2017). Pilon was used to correct the assembled contigs with Illumina reads (Walker et al., 2014). Annotation was carried out using RAST v2.0(Aziz et al., 2008) and BLASTp/BLASTn searches against the UniProtKB/SwissProt database (Boutet et al., 2016). The core genome alignment was performed using Roary and single nucleotide polymorphisms (SNPs) were extracted using snp-sites v2.3.2 (Page et al., 2015). Multilocus sequence typing (MLST) of strains, plasmid replicons, and ARGs were determined using the Center for Genomic Epidemiology^3^ web tools MLST v2.0, PlasmidFinder v2.1, and ResFinder v4.1, respectively. Insertion elements (IS) and integrons were annotated using online databases IS Finder (Siguier et al., 2006) and INTEGRALL (Moura et al., 2009). BRIG and Easyfig were employed to generate the genetic comparison figures (Alikhan et al., 2011; Sullivan et al., 2011).

### 2.4. Plasmid transferability and stability

Conjugation experiments were performed using broth-based method with the *E. coli* EC600 (rifampin-resistance) as the recipient, as described previously with minor modification (Li et al., 2022a). After the donors and recipients were grown to the exponential stage when the optical density at 600 nm (OD600) reaches ~0.5, mix them at a donor/recipient ratio of 1:1 before incubation at 37°C for 24 h. Transconjugants were selected on Luria-Bertani (LB) agar plates containing tigecycline (2 μg/mL) plus rifampin (400 μg/mL). The presence of *tet*(X4) in transconjugants was confirmed by PCR using the primers *tet*(X4)-F/R in Table 1.

The plasmid stability was studied by serial passage in antibiotic-free LB broth as previously described (Lv et al., 2020; Li et al., 2023b). Briefly, three separate cultures of *E. coli* transconjugants carrying the target plasmid were grown overnight in antibiotic-free LB broth, followed by dilution in fresh LB medium at a ratio of 1:10^2^. Serial passaging of the overnight culture to new LB broth was performed daily (approximately 10 generations of growth per passage), lasting for 14 days. 48 single clones of 14th passages were randomly selected from each culture, and the presence of *tet*(X4) was confirmed by PCR using primers *tet*(X4)-F/R.

### 2.5. Mutation analysis

The sequences of *ramR*, *oqxR*, and *rpsJ* in the tested strains were aligned with the reference sequence of tigecycline-susceptible isolate *K. pneumoniae* MGH 78578 (GenBank accession no. CP000647). The *tet*(A) variant was identified by comparing to the original *tet*(A) in plasmid pUUH239.2 (Accession no. NC_016966).

### 2.6. Real-time relative quantitative PCR assays

The RT-qPCR experiments were performed as previously described (He et al., 2015). Overnight bacterial cultures were diluted 1/100 into fresh LB broth and grown to the mid-exponential stage (OD600 ~ 0.5) at 37°C. The total bacterial RNA was harvested using a TaKaRa MiniBEST universal RNA extraction kit (TaKaRa, Dalian, Japan). The quantity and purity were evaluated using a NanoDrop 1,000 spectrophotometer (Thermo Scientific, Hvidovre, Denmark). RNA was reverse transcribed into cDNA using the PrimeScript RT reagent Kit with gDNA Eraser RR047A (TaKaRa, Dalian, China) according to the manufacturer’s instructions. RT-qPCR was performed using Taq Pro Universal SYBR qPCR Master Mix (Vazyme, Nanjing, China) on an FGD-96A real-time system (BIOER, Hangzhou, China) with 40 cycles of 30s at 95°C, 10s at 95°C, 30s at 60°C, 15 s at 95°C, 60s at 65°C, and 15 s at 97°C. Primers for the efflux pump genes (*acrB*, *acrE*, and *oqxB*) and the regulator genes (*ramA*, and *rarA*) were presented in Table 1. The relative expression levels were normalized against the 16 s rRNA gene, and the fold changes were calculated using the 2^-ΔΔCT^ method. A tigecycline-susceptible *K. pneumoniae* clinical isolate SCNJ10 (MIC ≤0.5 μg/mL) was used as a reference strain for the gene expression analysis. This experiment was repeated three times independently with triplicate samples. Data were analyzed with GraphPad Prism version 6.0 (GraphPad Software, San Diego, CA). Values returning a *p* value of *<*0.05 from a Student *t* test were taken as significant and indicated by an asterisk.

## 3. Results and discussion

### 3.1. Bacterial isolates and their phenotypic and genotypic resistance

During a survey evaluating the prevalence of tigecycline-resistant strains from the hospital sewage, a total of 113 tigecycline-resistant Enterobacterales isolates were collected, including 16 *E. coli*, 65 *Klebsiella* spp., 26 *Enterobacter* spp., and 6 *Citrobacter* spp., as revealed by 16 s rRNA gene analysis. Among these, six *E. coli* were positive for *tet*(X4), and three *K. pneumoniae* carried *tmexD* (Table 2). 14 strains were selected for further analysis in detail by whole genome sequencing (WGS) using the Illumina HiSeq platform, including six *tet*(X4)-bearing *E. coli*, three *tmexD-*bearing *K. pneumoniae*, and five additional tigecycline-resistant *K. pneumoniae* from different samples (Table 2), which all exhibited resistance to tigecycline with MICs of 8 to16 μg/mL. Of them, LZSFT34 (*E. coli*, carrying *tet*(X4)), LZSFT39 (*K. pneumoniae*, carrying *tmexCD-toprJ1*), LZSFZT33 (*E. coli*, carrying *tet*(X4)), and LZSRT11 (*K. pneumoniae*) were further sequenced using the Nanopore technology.

**Table 2:** Genomic characteristics of tigecycline-resistant strains.

Strain	Species	Antimicrobial resistance genes	Sequence type	Plasmid replicons	Antimicrobial resistance profile	Accession no.
LZSFT33	*E. coli*	*aadA1*, *aadA24*, *aadA2, bla*_TEM-1_, *tet*(X4), *tet*(A), *dfrA12*, *cmlA1*, *floR*, *qacL*, *sul3*, *qnrS1*	ST871	IncFIA(HI1), IncFII(pCoo), IncR, IncY	GEN-TET-TIG- CHL-SXT	JAVCAG000000000
LZSFT34	*E. coli*	*aph(3″)-Ib*, *aadA2*, *aph(6)-Id*, *floR*, *dfrA14*, *lnu*(F)*, qnrS1*, *sul2*, *tet*(X4), *tet*(A) ^a^	ST2144	Col(MP18), Col156, IncB/O/K/Z^a^, IncFIA	AMK-TET-CIP- TIG-CHL-SXT	CP132728-CP132735
LZSFZT29	*E. coli*	*aph(6)-Id, aph(3″)-Ib, aadA22, bla*_TEM-1_*, bla*_CMY-2_*, dfrA1, floR, qnrS1, qacE, tet*(X4), *tet*(A), *tet*(B), *lnu*(G), *mph*(A), *sul1, sul2*	ST88	IncB/O/K/Z, IncC, IncFIA(HI1), IncFIB, IncFIC(FII), IncHI1A, IncHI1B(R27), IncI1-I(Alpha)	TET-CIP-TIG-FOX-CHL-CTX- SXT	JAVCAB000000000
LZSFZT33	*E. coli*	*aph(6)-Id*, *aph(3″)-Ib*, *aadA22*, *bla*_CMY-2_, *floR*, *qnrS1*, *tet*(X4), *tet*(A), *tet*(B), *qacE*, *lnu*(G), *mph*(A), *sul1*, *sul2*	ST88	IncB/O/K/Z, IncC, IncFIA(HI1), IncFIB, IncFIC(FII), IncHI1A, IncHI1B(R27)	TET-CIP-TIG-FOX-CHL-CTX - SXT	CP132720-CP132725
LZSFZT27	*E. coli*	*aph(6)-Id*, *aph(3″)-Ib*, *aadA22*, *floR*, *tet*(X4), *tet*(A), *tet*(B), *qacE*, *lnu*(G), *mph*(A), *bla*_CMY-2_, *sul1*, *sul2*	ST88	IncB/O/K/Z, IncC, IncFIA(HI1), IncFIB, IncFIC(FII), IncHI1A, IncHI1B(R27)	TET-TIG-FOX- CHL-CTX-SXT	JAVCAC000000000
LZSRT46	*K. pneumoniae*	*aadA2*, *aac(3)-IId*, *aac(6′)-Ib*, *bla*_OXA-1_, *bla*_SHV-106_, *bla*_CTX-M-15_, *bla*_TEM-1_, *dfrA12*, *fosA6*, *oqxB20*, *oqxA6, sul1, mph*(A), *tet*(A)	ST15	IncFIB(K), IncFII(K)	AMK-GEN-TET-CIP-TIG-CTX	JAVCAF000000000
LZSFT31	*K. pneumoniae*	*aadA2*, *aac(3)-IId, bla*_CTX-M-55_*, bla*_SHV-172_*, catA2, dfrA12, fosA6, tet*(A)*, qnrS1, mph*(A)*, floR, oqxA5, oqxB19, sul1, sul2*	ST3179	IncR	GEN-TET-CIP-TIG-FOX-CHL-CTX-SXT	JAVCAH000000000
LZSFZT3	*K. pneumoniae*	*aac(6′)-Ib*, *aadA16*, *aph(3′)-Ia*, *arr-3*, *bla*_NDM-1_, *ble-MBL*, *bla*_SHV-187_, *dfrA27*, *tet*(A), *fosA*, *floR*, *bla*_TEM-1_, *bla*_CTX-M-3_*, sul1*, *mph*(A), *qnrS1*, *oqxB19*, *oqxA6*	ST1574	IncFII(K), IncN2, repB(R1701)	TET-CIP-TIG-FOX-CHL-CTX-MEM-SXT	JAVCAE000000000
LZSRT3	*K. pneumoniae*	*aac(6′)-Ib-cr*, *aph(6)-Id*, *aac(3)-IV*, *aph(3″)-Ib, armA*, *aadA2b, aph(3′)-Ia*, *aadA16*, *aac(3)-IId*, *aph(4)-Ia*, *aadA1*, *arr-3*, *cmlA1*, *floR*, *fosA, qnrB4*, *oqxA*, *oqxB*, *qacL*, *qacE*, *bla*_DHA-1_*, bla*_SHV-12_*, tet*(A)*, dfrA27, mph*(E)*, msr*(E)*, mph*(A)*, sul1, sul3*	NT*	IncR, IncHI1B, IncFIB(K)	AMK-GEN-TET- CIP-TIG-CHL-FOX-CTX-SXT	JAVCAD000000000
LZSFT39	*K. pneumoniae*	*aac(6′)-Ib-cr*, *aph(3′)-VI*, *aadA5*, *aac(6′)-Ib3*, *dfrA1^a^*, *oqxA*, *oqxB*, *qacE^a^*, *arr-3*, *fosA3*, *fosA*, *bla*_DHA-1_, *bla*_NDM-1_, *tet*(A)*^a^*, *qnrS1^a^*, *mph*(A), *sul1^a^*	ST1306	Col440I, IncFIA(HI1), IncFIB(K), IncFIB(pQil), IncR, IncU	AMK-GEN-TET- CIP-TIG-FOX-CTX-MEM-SXT	CP132736-CP132740
LZSRT11	*K. pneumoniae*	*aac(6′)-Ib-cr*, *aac(3)-IId*, *aadA2*, *bla*_SHV-28_, *bla*_SHV-106_, *bla*_OXA-1_, *bla*_TEM-1_, *bla*_CTX-M-15_, *dfrA12*, *tet*(A), *oqxA*, *oqxB*, *mph*(A), *fosA*, *catB3*, *qacE*, *sul1*	ST15	IncFIB(K), IncFII(K)	AMK-GEN-TET- CIP-TIG-CTX - SXT	CP132726, CP132727

^a^≥two copies; NT* novel type (gapA-infB-mdh-pgi-phoE-rpoB-tonB, 2–9–2-1-13-1-31) closely matches ST1228. AMK, amikacin; GEN, gentamicin; TET, tetracycline; CIP, ciprofloxacin; TIG, tigecycline; FOX, cefoxitin; CHL, chloramphenicol; CTX, cefotaxime; MEM, meropenem; SXT, sulfamethoxazole-trimethoprim.

According to the WGS data, three strains were identified as redundant (i.e., isolated from the same location, belonging to the same species and the same sequence type [ST], and carrying the same ARGs and plasmid replicons). We excluded these redundant isolates, leaving 11 isolates, including five *E. coli* strains (LZSFT34, LZSFZT27, LZSFZT29, LZSFZT33, and LZSFT33), and six *K. pneumoniae* (LZSFT39, LZSRT11, LZSRT46, LZSFT31, LZSFZT3, and LZSRT3) (Table 2). Antimicrobial susceptibility testing showed that most of these tigecycline-resistant strains exhibited co-resistance to tetracycline (*n* = 11), cefotaxime (*n* = 9), ciprofloxacin (*n* = 9), and sulfamethoxazole-trimethoprim (*n* = 10). Some of them were also resistant to chloramphenicol (*n* = 8), cefoxitin (*n* = 7), or gentamicin (*n* = 6). Only three strains (LZSRT11, LZSRT46, and LZSRT3) showed resistance to amikacin. Alarmingly, two isolates (LZSFT39, and LZSFZT3) were also resistant to meropenem. Genome analysis showed that 63 different ARGs were detected in the 11 tigecycline-resistant strains, and each of them carried 10 to 29 ARGs (Table 2). Thirteen types of β-lactam resistance genes were detected, including *bla*_CTX-M-15_, *bla*_CTX-M-55_*, bla*_CMY-2_, and *bla*_SHV-12_ that were reported to be common ESBL genes in clinical isolates (Zhang et al., 2013, 2014; Feng et al., 2015). Of note, the carbapenem resistance gene *bla*_NDM-1_ was found in two strains (LZSFZT3 and LZSFT39). The coexistence of *bla*_NDM-1_ and tigecyclineresistance determinant *tmexCD-toprJ* would pose a serious threat to the treatment of complicated infections by multidrug-resistant (MDR) Gram-negative bacterial infections.

### 3.2. Genomic characteristics of *tet*(X4)-bearing *Escherichia coli* isolates

**Figure 1:**
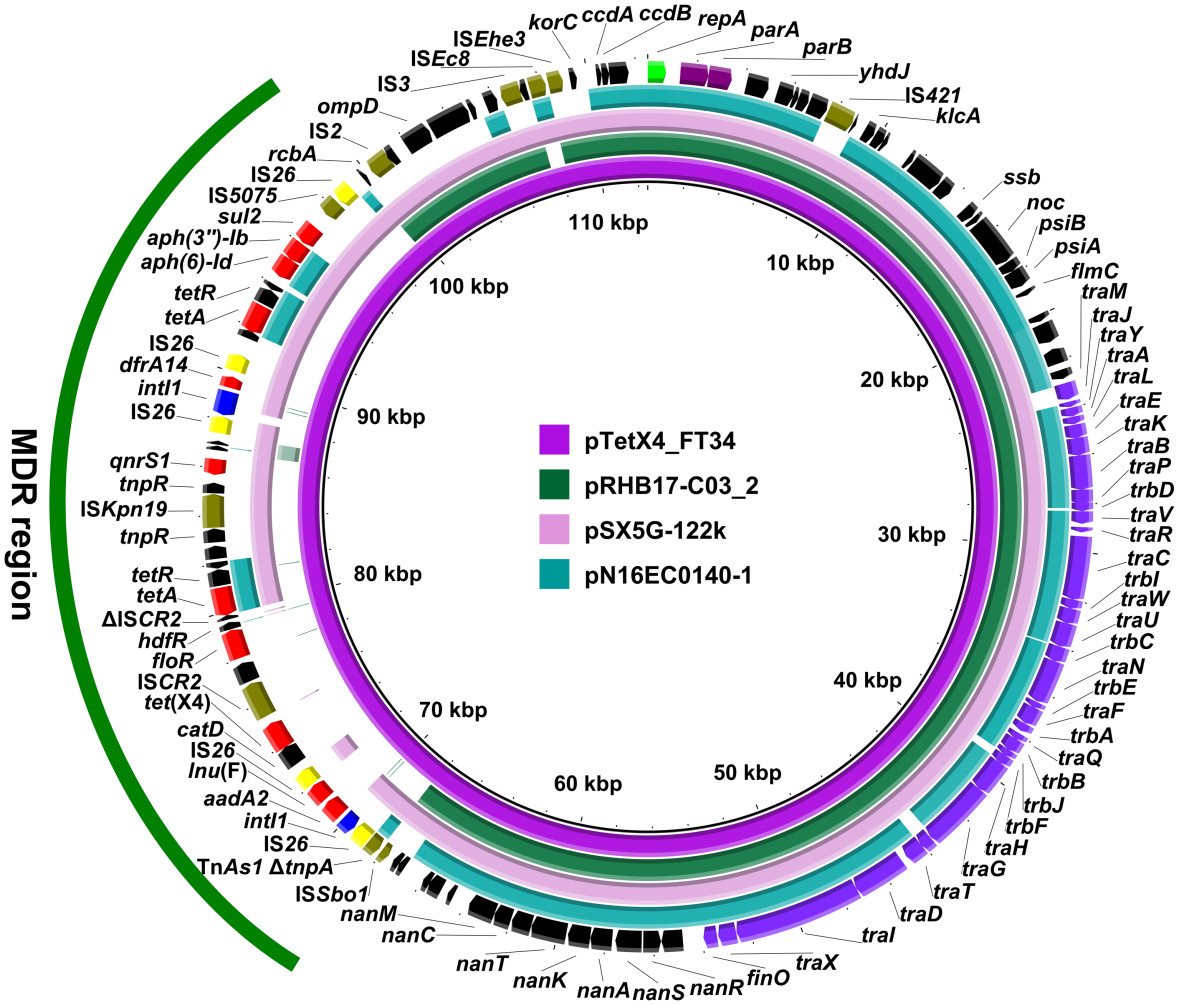
Circular comparison of pTetX4_FT34 with related plasmids. The complete sequence of pTetX4_FT34 was used as the reference. Arrows on the outer ring indicate deduced ORFs and their orientations. Genes for replication (*repA*), maintenance (*parAB*), and conjugal transfer (*tra*, *trb* genes) are indicated in green, purple, and violet. The accessory resistance region is indicated by green curve. Genes for resistance, integrase, IS*26*, and other transposase genes are highlighted in red, blue, yellow, and olive, respectively.

Strain LZSFT34 consists of a 4,945,961-bp chromosome with a GC content of 50.93%, and seven plasmids ranging in size from 1,166 to 114,852 bp. It has 10 ARGs mediating resistance to aminoglycosides (*aph(3″)-Ib*, *aadA2*, and *aph(6)-Id*), trimethoprim (*dfrA1*), quinolones (*qnrS1*), phenicol (*floR*), sulfonamides (*sul2*), tetracyclines (*tet*(A), two copies), lincosamide (*lnu*(F)), and tigecycline (*tet*(X4)). All these ARGs are located on the *tet*(X4)-bearing plasmid pTetX4_FT34, suggesting that tigecycline resistance could be co-selected by other antimicrobial resistance determinants. pTetX4_FT34 is 112,510 bp in size with an average GC content of 51.75% and belongs to the IncFIA group. The plasmid backbone was composed of regions for replication (*repA*), maintenance (*parAB*), and conjugal transfer (*tra, trb* genes) (Figure 1). It also carried a ~ 31 kb accessory resistance region, which contained all the ARGs and abundant mobile genetic elements that are responsible for the formation of this mosaic MDR region. BLASTn comparison of pTetX4_FT34 with plasmids in the NCBI database showed that it is most similar (91% coverage, 99.96% nucleotide identity) to pSX5G-122 k (MZ367885, *E. coli*, pork, China), and partial similar (>60% coverage, >99.9% identity) to pRHB17-C03_2 (CP057706, *E. coli*, pig, United Kingdom) and pN16EC0140–1 (CP043748, *E. coli*, pork, United States). Of note, *tet*(X4) is absent from these similar plasmids, indicating a stepwise integration of horizontally acquired *tet*(X4) in pTetX4_FT34.

**Figure 2:**
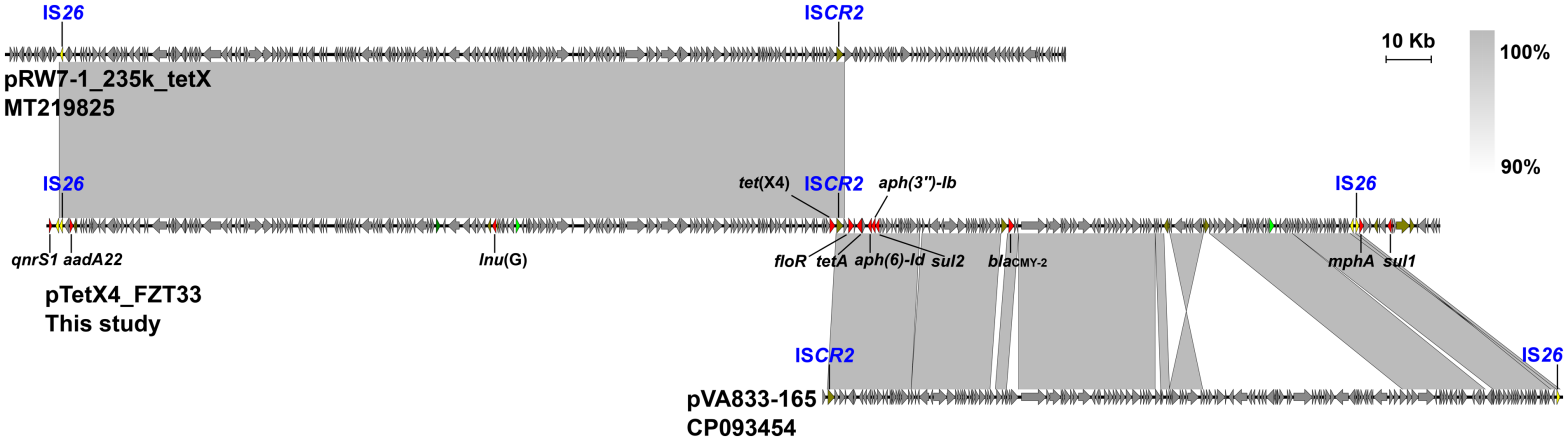
Organization of pTetX4_FZT33, and comparisons to related plasmids. Genes are denoted by arrows. ARGs, replication genes, IS*26*, and other transposase genes are indicated in red, green, yellow, and olive, respectively. The presumed recombination junctions (IS*CR2* and IS*26*) are highlighted. Regions of >90% homology are indicated by grey shading.

According to the draft genome sequences, *E. coli* LZSFZT27, LZSFZT29, and LZSFZT33 are all assigned to ST88 by MLST analysis. While they are different strains, with at least 258 SNPs between each other in their core genomes. LZSFZT33 was selected as a representative strain and was further sequenced using Nanopore to obtain the whole-genome sequences. According to the WGS data, LZSFZT33 has a 5,056,393-bp chromosome with an average GC content of 50.69%, four plasmids (5,058 to 127,405 bp), and one unclosed contig. Fourteen ARGs were identified in LZSFZT33, including *aph(6)-Id*, *aph(3″)-Ib*, *aadA22*, *bla*_CMY-2_, *floR*, *qnrS1*, *tet*(X4), *tet*(A), *tet*(B), *qacE*, *lnu*(G), *mph*(A), *sul1*, and *sul2*. All of them are distributed on the unclosed contig (designated pTetX4_FZT33), except for the chromosomally located *tet*(B). The pTetX4_FZT33 is 309,872 bp in length with an average GC content of 48.71%. It is a novel hybrid structure that contains four different replicons, IncC, IncFIA(HI1), IncHI1A, and IncHI1B(R27). Sequence analysis showed that pTetX4_FZT33 consists of a 175-kb region (2,871 to 177,389 bp) which is almost identical (99.95% identity) to pRW7-1_235k (MT219825, *E. coli*, wastewater, China), and a 30-kb region (216,124 to 246,496 bp) that is highly similar (99.99% identity) to IncC type plasmid pVA833-165 (CP093454, *K. pneumoniae*, patient, Chile) (Figure 2). According to the genetic organization, IS*CR2* downstream of *tet*(X4), IS*26* upstream of *mphA*, and IS*26* downstream of *qnrS1* are most likely to be the recombination junctions of the hybrid plasmid (Figure 2).

**Figure 3:**
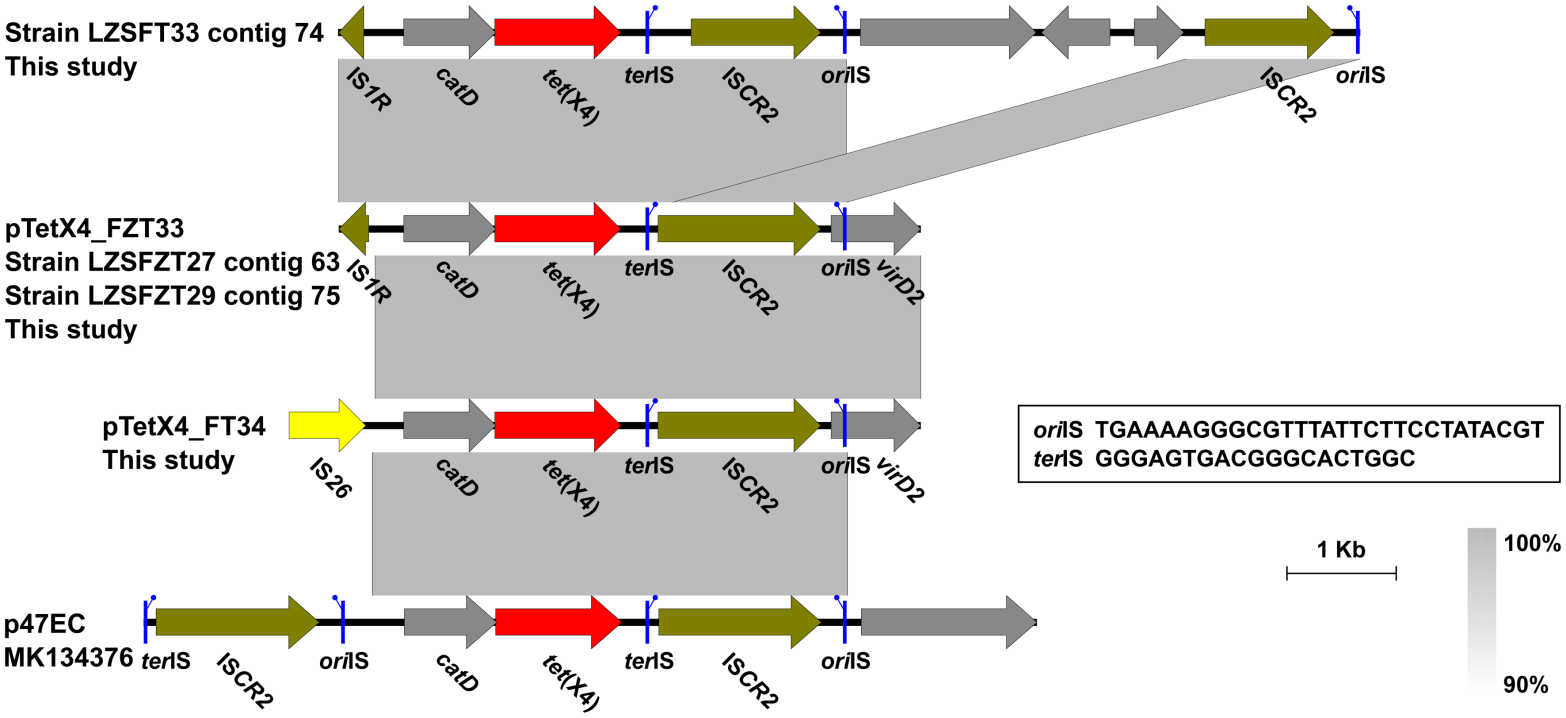
Genetic contexts of the *tet*(X4) in isolates from this study and the reported plasmid p47EC. Genes are denoted by arrows. ARGs, IS*26*, and other transposase genes are indicated in red, yellow, and olive, respectively. The putative *ori*IS (left-facing sticks in blue) and *ter*IS (right-facing sticks in blue) are marked. Regions of >90% homology are indicated by grey shading. Δ represents truncated genes.

It has been known that IS*CR2* plays a vital role in *tet*(X4) transmission by rolling-circle transposition (Fang et al., 2020). In this study, an intact IS*CR2* was found downstream of the *catD-tet*(X4) cassette, leaving the structure *catD-tet*(X4)-*terlS*-IS*CR2*-*orilS* (Figure 3), which was the reported *tet*(X4)-bearing circular intermediate (He et al., 2019). Instead of another IS*CR2* located upstream of the *catD*-*tet*(X4)-IS*CR2* cassette, as had been reported previously in other plasmids (He et al., 2019), an IS*1R* or IS*26* was identified in our cases. A similar structure was also identified in IncHI1 and IncX1 type plasmids from animal-derived *E. coli* strains (Yu et al., 2021). The findings highlight the diversity of *tet*(X4)-positive plasmids and *tet*(X4)-bearing genetic contexts in *E. coli* clones from different ecological niches.

Further conjugation assays showed that *tet*(X4) could be successfully transferred from *E. coli* LZSFZT27, LZSFZT29, and LZSFZT33 to laboratory strain *E. coli* EC600, leading to an increased MIC of tigecycline in EC600 by 16-fold (from 0.5 to 8 μg/mL). This result indicates that *tet*(X4)-bearing plasmids in these three strains were self-transmissible. However, the acquisition of *tet*(X4) from LZSFT33 and LZSFT34 failed to confer tigecycline resistance in EC600, despite that a 4-fold (0.5 versus 2 μg/mL) increase in MIC of tigecycline was detected. After 14d (approximately 140 generations) of serial passage without antibiotic treatment, *tet*(X4)-bearing plasmids from LZSFZT27, LZSFZT29, LZSFZT33, LZSFT33 and LZSFT34 were all stably maintained in the transconjugants host, with 93.8–100% retention. The transferability and stability of plasmids containing *tet*(X4) have serious public health implications.

### 3.3. Characterization of tigecycline-resistant *Klebsiella pneumoniae* strains

**Figure 4:**
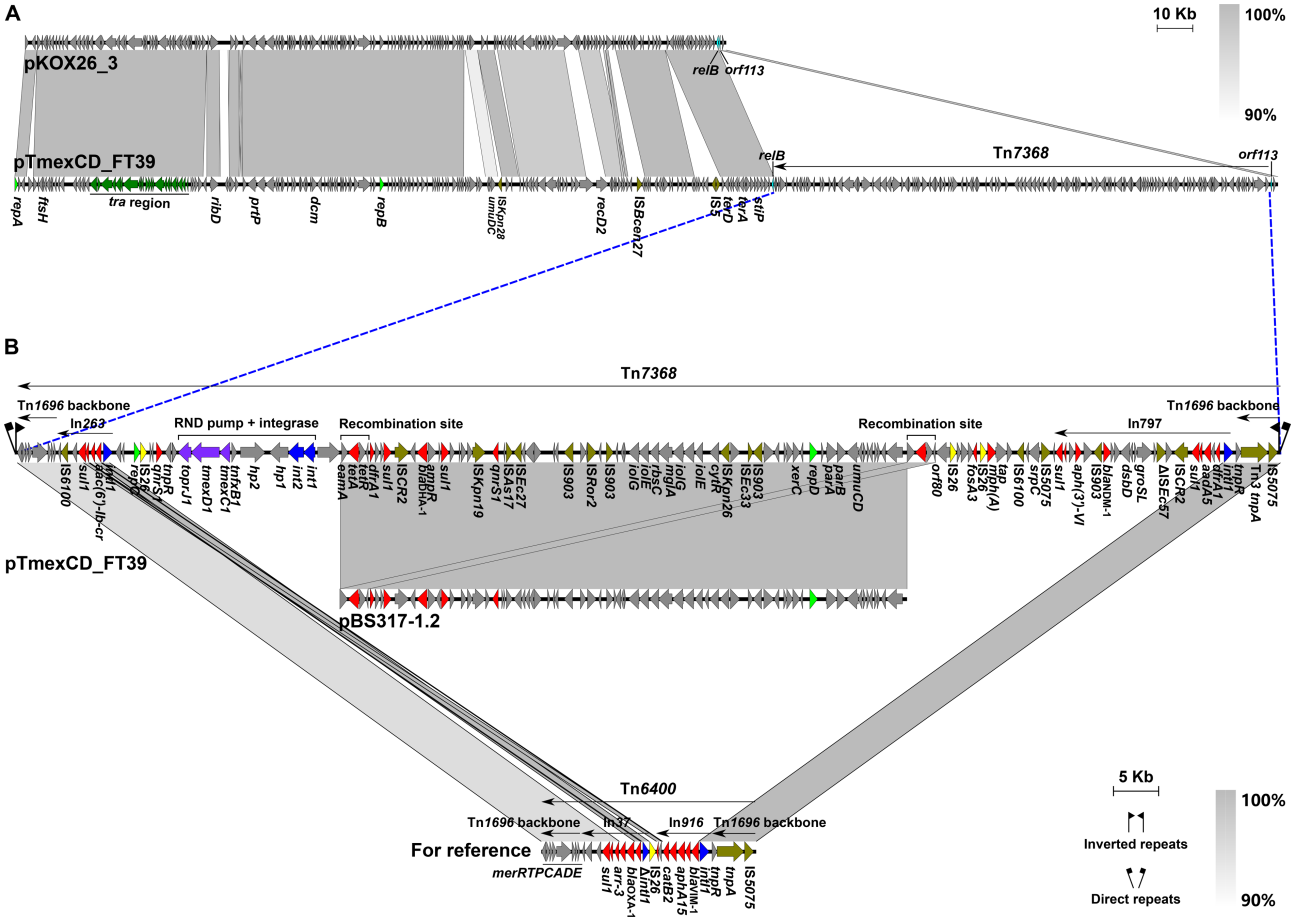
Genetic features of pTmexCD_FT39. **(A)** Comparison of pTmexCD_FT39 with pKOX26_3. The novel transposon Tn*7368* was inserted into a site between *relB* and *orf113.* **(B)** Organization of Tn*7368*, and comparisons to related regions. Genes are denoted by arrows. ARGs, integrase genes, replication genes, IS*26*, and other transposase genes are indicated in red, green, yellow, and olive, respectively. The *tra* region is highlighted in dark green, and the *tmexCD1-toprJ1* gene cluster is in purple. Regions of >90% homology are indicated by grey shading. Δ represents truncated genes.

Two non-redundant tigecycline-resistant *K. pneumoniae* LZSFT39 and LZSRT3 were identified to carry the *tmexCD1-toprJ1*. LZSFT39 contains a chromosome of 5,138,005 bp (GC content of ~57.57%), and four plasmids ranging in size from 13,871 to 355,922 bp. LZSFT39 carries 17 different ARGs, including *aac(6′)-Ib-cr*, *aph(3′)-VI*, *aadA5*, *aac(6′)-Ib3*, *dfrA1* (two copies), *oqxA*, *oqxB*, *qacE* (five copies), *arr-3*, *fosA3*, *fosA*, *bla*_DHA-1_, *bla*_NDM-1_, *tet*(A) (two copies), *qnrS1* (two copies), *mph(A)*, *sul1* (five copies). Of them, *fosA*, *oqxA*, and *oqxB* were located on the chromosome, while the remaining ARGs were all distributed on the *tmexCD1-toprJ1*-bearing plasmid pTmexCD-FT39. It is an IncR/IncU hybrid plasmid, with 355,922-bp in length and an average GC content of 49.38%. BLASTn analysis showed that pTmexCD-FT39 is most similar (77% coverage, 99.87% identity) to p7_SCLZS62, a *tmexCD1-toprJ1*-bearing plasmid from *Raoultella planticola* isolated from the same sample collection site in November 2019 (Li et al., 2022b). This finding highlights the dissemination of tigecycline resistance mediated by plasmids between bacterial communities in hospital sewage. Sequence analysis showed that the backbone of pTmexCD-FT39 (nt 1 to 213,922 bp and 353,771 to 355,922 bp) is highly similar (86% coverage, 99.7% identity) to pKOX26_3 (CP089402, *Klebsiella oxytoca*, patient, Australia), and its accessory region that contained all the ARGs and *tmexCD1-toprJ1* gene cluster was inserted into a site between *relB* and *orf113* of pKOX26_3, revealing that pTmexCD-FT39 is likely derived from a pKOX26_3-like plasmid (Figure 4A).

We further found that the accessory region was a novel MDR transposon that was designated Tn*7368*, according to the nomenclature of transposons.^4^ Tn*7368* is 139,848 bp (corresponding to bases 213,923 to 353,770 in GenBank accession number CP132737) with an average GC content of 55.56%, which differs from that of the rest of the plasmid (GC content, ~45.37%). It was identified as Tn*6400*-derivative with similar *tnpAR* (99.97% identity) and *mer* (90.70% identity) modules, and was bracketed by 5-bp direct repeats (DRs, TTTCA) (Figure 4B). Tn*7368* is a mosaic structure composed of multiple class I transposons (such as In797 and In263) and insertion sequences (such as IS*26* and IS*6100*), and it carries the *int1-int2-hp1-hp2*-*tnfxB1*-*tmexCD1*-*toprJ1* core genetic structure as described in reference plasmid pHNAH8I-1 (MK347425) (Lv et al., 2020). Of note, a 62.6-kb region (nt 249,836 to 312,442 bp) inside Tn7368 is almost identical (>99.9% identity) to the IncR-type plasmid pBS317-1.2 (CP063938) that was found in a *K. pneumoniae* isolate from the human fecal sample in China. Sequence analysis reveals that homologous recombination mediated by the 2,983-bp *eamA*-*tet(A)*-*tetR-orf80* module is most likely to contribute to the formation of such a structure (Figure 4B).

**Figure 5:**
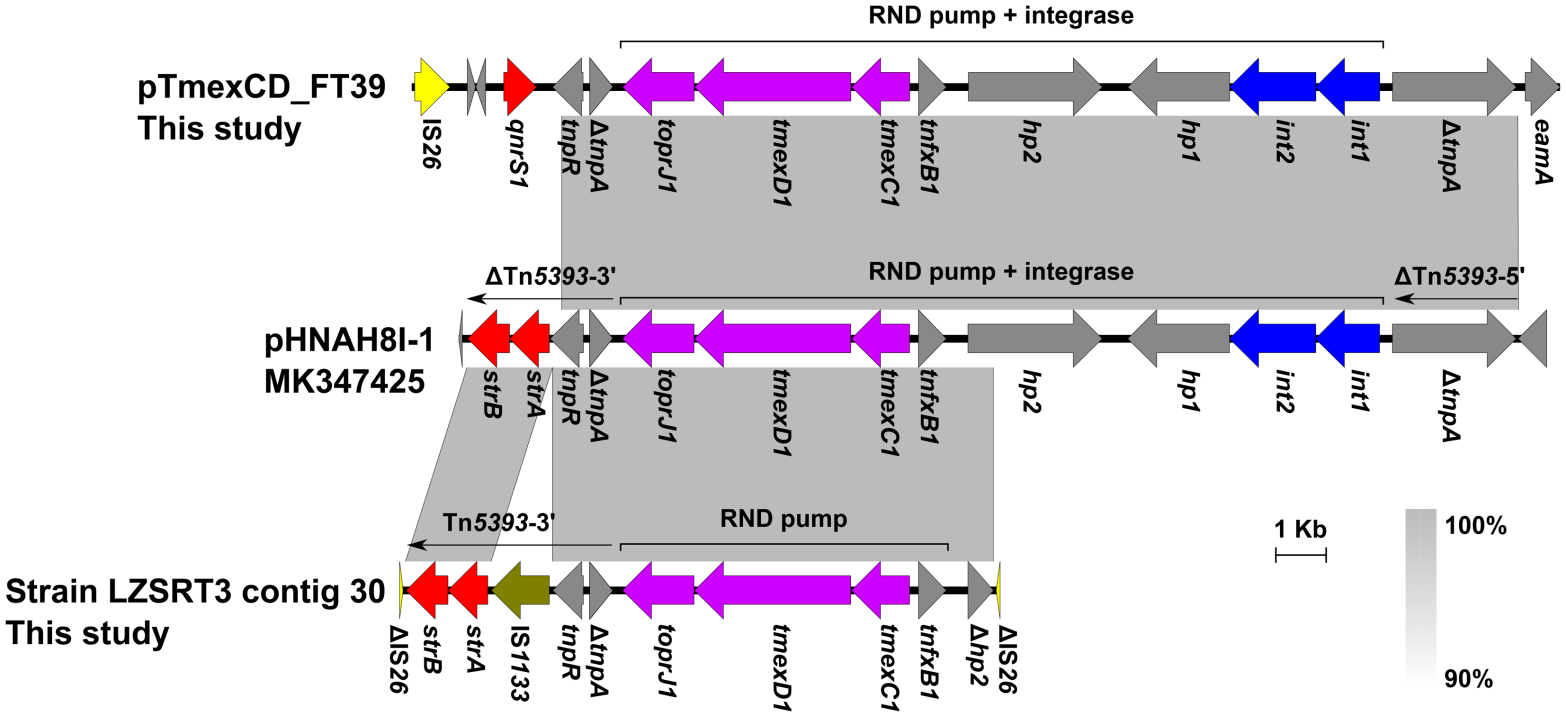
Genetic contexts of the *tmexCD1*-*toprJ1* in isolates from this study and the reported plasmid pHNAH8I-1. Genes are denoted by arrows. ARGs, integrase genes, IS*26*, and IS*1133* are indicated in red, blue, yellow, and olive, respectively. The *tmexCD1-toprJ1* gene cluster is highlighted in purple. Regions of >90% homology are indicated by grey shading. Δ represents truncated genes.

In LZSRT3, the *tmexCD1*-*toprJ1* genes coexist with 29 ARGs, including *aac(6′)-Ib-cr*, *aph(6)-Id*, *aac(3)-IV*, *aph(3″)-Ib*, *armA*, *aadA2b*, *aph(3′)-Ia*, *aadA16*, *aac(3)-IId*, *aph(4)-Ia*, *aadA1*, *arr-3*, *cmlA1*, *floR*, *fosA*, *qnrB4*, *oqxA*, *oqxB*, *qacL*, *qacE*, *bla*_DHA-1_, *bla*_SHV-12_, *tet*(A), *dfrA27*, *mph*(E), *msr*(E), *mph*(A), *sul1*, and *sul3*. It has been suggested that site-specific integrases and Tn*5393*-like transposon are responsible for the capture and transmission of *tmexCD1-toprJ1* in *Klebsiella* (Dong et al., 2022). In LZSRT3, the *tnfxB1*-*tmexCD1-toprJ1* gene cluster was identical to that in the pHNAH8I-1, while the *int1-int2-hp1* and the adjacent ΔTn*5393*-5′ was lost, which was most likely to be caused by the recombination event of IS*26* that was inserted into *hp2* (Figure 5). Of note, compared to the Tn*5393* residue downstream of the *tmexCD1-toprJ1* gene cluster in pHNAH8I-1, LZSRT3 had intact Tn*5393*-3′. Unlike the scenario in LZSRT3, the *int1-int2-hp1-hp2*-*tnfxB1*-*tmexCD1*-*toprJ1* segment was intact in strain LZSFT39, while the ΔTn*5393-*3′ was truncated and replaced by the Δ*tnpA*-*tnpR*-*qnrS1*-*IS26* module (Figure 5). The findings suggest a parallel diversification and evolution of *tmexCD1-toprJ1*-bearing genetic contexts in *K. pneumoniae*. The transfer ability of *tmexCD1-toprJ1*-bearing plasmids was not determined because their host strains LZSFT39 and LZSRT3 were highly resistant to sodium azide (MIC ≥300 μg/mL, used for selection of *E.coli* J53) and rifampicin (MIC ≥1,000 μg/mL, used for selection of *E.coli* EC600). Given that the backbone of pTmexCD-FT39 is almost identical (99.87% identity) to p7_SCLZS62, which was previously identified to be self-transmissible (Li et al., 2022b), the transfer of pTmexCD-FT39 seems likely.

LZSRT11 comprises a 5,266,515-bp chromosome (GC content, ~57.47%), and one 221,089-bp plasmid p1_RT11. In LZSRT11, ARGs including *oqxA*, *oqxB*, *fosA*, *bla*_SHV-28_, *bla*_SHV-106_, and *aac(3)-IId* are located on the chromosome, while *aac(6′)-Ib-cr*, *aadA2*, *bla*_OXA-1_, *bla*_TEM-1_, *bla*_CTX-M-15_, *dfrA12*, *tet*(A), *mph*(A), *catB3*, *qacE*, and *sul1* are distributed on the p1_RT11. This plasmid has two replicons, IncFIB(K) and IncFII(K). BLASTn analysis showed that it is highly similar (>90% coverage, >99% identity) to several plasmids from clinical *K. pneumoniae* isolates in China (Figure S1), such as pXHKP75-1 (CP066896, Shanghai), p1_CRKP_11(CP107469, Wuhan), and pC2660-2 (CP039809, Beijing), revealing the circulation and transmission of this MDR plasmid across China. Of note, we found that LZSRT11 as well as LZSRT46 belongs to ST15, which is a high-risk clone with frequent hospital outbreaks in China and has emerged carrying virulence-resistant heterozygous plasmids associated with carbapenemases and ESBL genes (Huang et al., 2022; Zhao et al., 2022). The identification of ST15 tigecycline-resistant *K. pneumoniae* highlights that close surveillance is urgently needed to monitor the prevalence of ST15 *K. pneumoniae* in the local clinical settings.

We did not identify *tmexCD-toprJ* or any genes belonging to the *tet*(X) family in LZSRT11 and the remaining three tigecycline-resistant *K. pneumoniae* strains (LZSRT46, LZSFT31, and LZSFZT3). It has been suggested that tigecycline resistance was mainly caused by mutations in *ramR* or *oxqR* and the associated overexpression of efflux pumps in *K. pneumoniae* (He et al., 2015). To confirm this, mRNA expression and sequences of several related genes were analyzed. All the strains contained mutations in *ramR*, among which LZSFZT3 had a frameshift mutation and the remaining five strains had point substitutions (Table 3). Consistent with the finding by a previous study that not all of the changes within *ramR* resulted in *ramA* overexpression (Rosenblum et al., 2011), upregulation of *ramA* was only identified in strain LZSRT46 in this study (Table 3). Besides, the upregulated *ramA* did not lead to a higher expression level of *acrB* in LZSRT46. And, strain LZSFZT3 had an increased expression level of *acrB* but with baseline expression of *ramA*. These results suggest that the expression level of the *ramA* was not always correlated with that of the *acrB* gene. Only one isolate, LZSRT3, harbored a point mutation (V130A) in the *oqxR* gene (Table 3). The V130A mutation had been identified to be associated with increased transcript level of *rarA* and accounted for *oqxAB* overexpression (Cheng et al., 2020). In LZSRT3, the overexpression of *oqxAB* was also observed but the transcription of *rarA* was not significantly changed. The findings highlight that further research is needed to clarify the regulatory networks involved in tigecycline resistance in *K. pneumoniae* (He et al., 2015).

**Table 3:** Tigecycline resistance mechanisms of *Klebsiella pneumoniae* strains.

Strain	MIC (μg/ml)^a^	Relative mRNA expression^b^					Presence of *tmexCD-toprJ*	Mutation		
		*ramA*	*acrB*	*rarA*	*oqxB*	*acrE*		*ramR*	*oqxR*	*tet*(A)
SCNJ10	0.5	1.24 ± 0.92	1.01 ± 0.22	1.9 ± 2.68	1.78 ± 2.31	1.94 ± 1.61				
LZSFT39	16	1.24 ± 1.28	0.27 ± 0.02	0.18 ± 0.04	1.81 ± 0.75	0.73 ± 0.42	**+**	K194 Stop;		542 deletion^d^
LZSRT3	16	1.14 ± 1.22	1.58 ± 1.2	0.2 ± 0.16	48.59 ± 7.82*	0.66 ± 0.77	**+**	A19V; K194 Stop;	V130A	
LZSRT11	8	0.48 ± 0.36	0.3 ± 0.18	0.51 ± 0.48	2.52 ± 0.92	0.75 ± 0.61		A19V; K194Stop		
LZSRT46	16	151.27 ± 21.46*	1.98 ± 2.03	0.72 ± 0.25	19.96 ± 18.43	1.59 ± 1.78		A19V; Q122Stop		
LZSFT31	8	292.03 ± 234.8	1.95 ± 1.01	1.03 ± 0.93	1169.68 ± 1568.77	55.55 ± 82.08		W89C; K194Stop		
LZSFZT3	16	1185.56 ± 1098.49	8.03 ± 0.81*	0.82 ± 0.46	149.49 ± 109.85	70.81 ± 111.82		114 deletion^c^		G300E Deletion (28 bp)^e^

^a^Tigecycline MIC. ^b^Relative expression compared with tigecycline-susceptible *K. pneumoniae* strain SCNJ10. Results represent the means of three runs ± standard deviation. *Genes that are significantly upregulated (*p* < 0.05). ^c^Nucleotide position 114 (G) of the ramR locus of LZSFZT3 was deleted. The resulting coding sequence led to a frameshifted RamR protein. ^d^Nucleotide position 542 (T) of the tet(A) locus of LZSFT39 was deleted. The resulting coding sequence led to a frameshifted Tet(A) protein. ^e^Twenty-eight nucleotides were deleted at the 3′ end of the tet(A) locus of LZSFZT3 and this led to an abnormal Tet(A) protein.

A previous study suggested that the increased expression of *acrEF* plays a role in tigecycline resistance in *K. pneumoniae* (Li et al., 2023a). However, no strains displayed overexpression of *acrE* in this study. Also, the *rpsJ* mutation was not detected in all strains. In addition, LZSFT39 and LZSFZT3 had mutations of *tet*(A) that have not been reported before. Whether these variants *tet*(A) contribute to tigecycline resistance warrants further study. In all, *tmexCD1*-*toprJ1* and overexpression of efflux pump *oqxAB* or *acrAB* caused by mutations in *oxqR* or *ramR* may explain the tigecycline resistance in LZSFT39, LZSRT3, and LZSFZT3. While, for strains LZSRT11, LZSRT46, and LZSFT31, novel alternative mechanisms are presumed to exist.

## 4. Conclusion

In summary, to our knowledge, this is the first detailed report to describe the genome characteristics of tigecycline-resistant *E. coli* and *K. pneumoniae* from hospital sewage. Our study revealed novel hybrid plasmids and transposon in the dissemination of *tet*(X4) or *tmexCD1-toprJ1* and also provided insight into the *oqxAB*/*acrAB*-encoding tigecycline resistance. Considering the potential threats of the tigecycline resistance genes to public health, continuous monitoring is needed to understand their evolution and transmissible pathways of these high-risk genes. Further researches are also required to investigate the epidemiological links between resistant isolates from the natural environment and humans.

## Data availability statement

The datasets presented in this study can be found in online repositories. The names of the repository/repositories and accession number(s) can be found at: https://www.ncbi.nlm.nih.gov/genbank/, PRJNA1004410 https://www.ncbi.nlm.nih.gov/genbank/, CP132725 https://www.ncbi.nlm.nih.gov/genbank/, CP132730 https://www.ncbi.nlm.nih.gov/genbank/, CP132737 https://www.ncbi.nlm.nih.gov/genbank/, CP132727.

## Author contributions

YL: Conceptualization, Formal analysis, Writing – original draft. YF: Formal analysis, Methodology, Resources, Writing – review & editing. YQ: Formal analysis, Methodology, Resources, Writing – review & editing. QL: Resources, Software, Writing – review & editing. MY: Formal analysis, Methodology, Resources, Writing – review & editing. LZ: Conceptualization, Supervision, Writing – review & editing.

## Footnotes

^1^https://blast.ncbi.nlm.nih.gov/Blast.cgi

^2^http://www.eucast.org/clinical_breakpoints/

^3^http://www.genomicepidemiology.org/

^4^http://transposon.lstmed.ac.uk/
