# Supplementary material for: Genomic characterization of tigecycline-resistant Escherichia coli and Klebsiella pneumoniae isolates from hospital sewage
Source: Front Microbiol. 2023 Nov 10;14:1282988. doi: 10.3389/fmicb.2023.1282988 (PMC10667442; doi:10.3389/fmicb.2023.1282988)
Supplement: Supplementary file 1 [file Data_Sheet_1.DOCX]

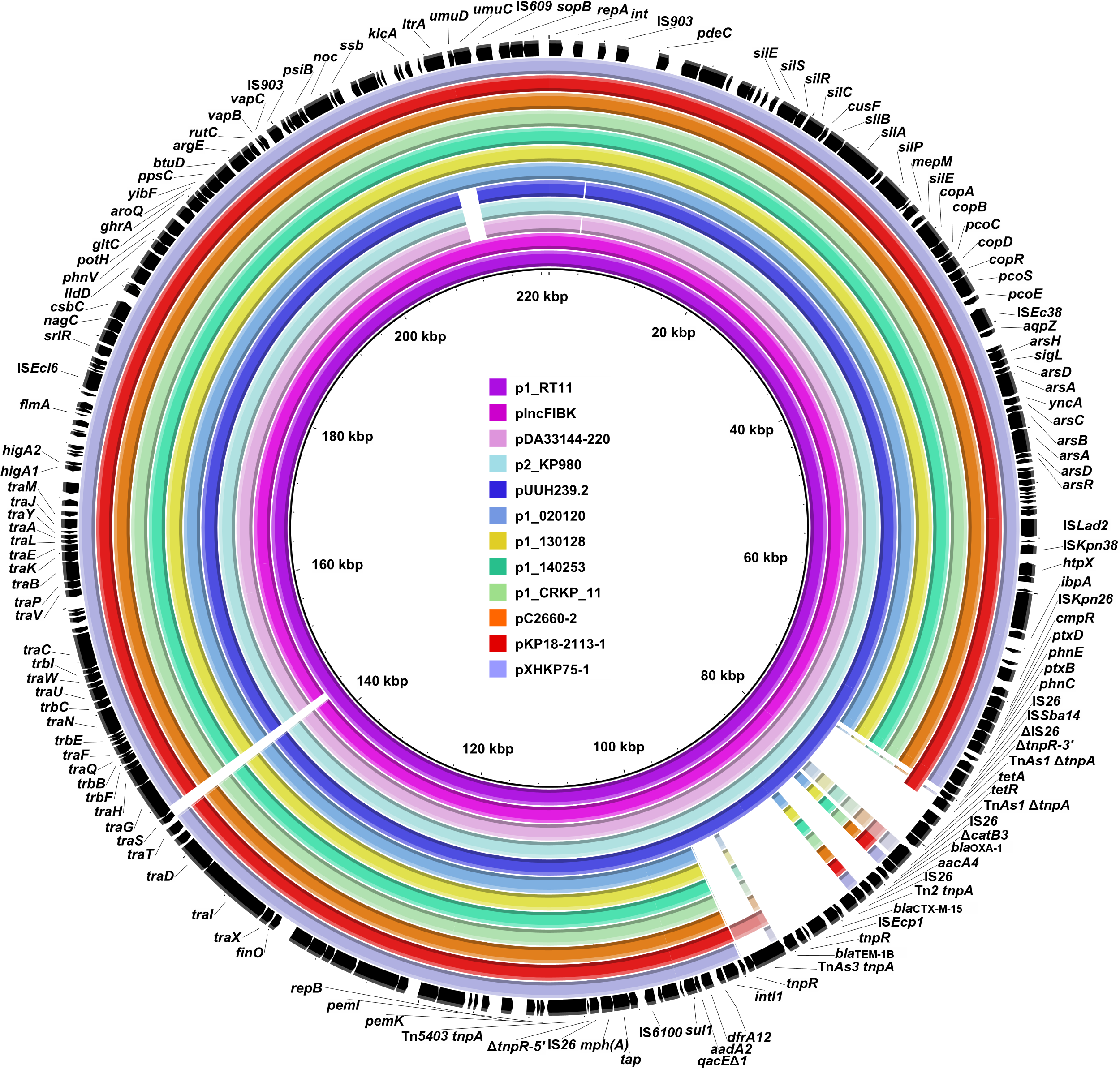


**Figure S1** **Circular comparison of p1_RT11 with related plasmids.** The complete sequence of p1_RT11 was used as the reference. Arrows on the outer ring indicate deduced ORFs and their orientations. Accession numbers for the plasmids from inner to outer ring are p1_RT11 (CP132727), pIncFIBK (CP036188), pDA33144-220 (CP029591), p2_KP980 (LR025089), pUUH239.2 (CP002474), p1_020120 (CP043359), p1_130128 (CP127237), p1_140253 (CP097628), p1_CRKP_11 (CP107469), pC2660-2 (CP039809), pKP18-2113-1 (CP082031), pXHKP75-1 (CP066896).
